# Muscarinic Receptor Modulation of the Cerebellar Interpositus Nucleus In Vitro

**DOI:** 10.1007/s11064-018-2613-9

**Published:** 2018-08-16

**Authors:** J. Pickford, R. Apps, Z. I. Bashir

**Affiliations:** School of Physiology, Pharmacology and Neuroscience, University of Bristol, Bristol, BS8 1TD UK

**Keywords:** Cerebellar nuclei, Acetylcholine, Muscarinic receptors, Carbachol

## Abstract

How the cerebellum carries out its functions is not clear, even for its established roles in motor control. In particular, little is known about how the cerebellar nuclei (CN) integrate their synaptic and neuromodulatory inputs to generate cerebellar output. CN neurons receive inhibitory inputs from Purkinje cells, excitatory inputs from mossy fibre and climbing fibre collaterals, as well as a variety of neuromodulatory inputs, including cholinergic inputs. In this study we tested how activation of acetylcholine receptors modulated firing rate, intrinsic properties and synaptic transmission in the CN. Using in vitro whole-cell patch clamp recordings from neurons in the interpositus nucleus, the acetylcholine receptor agonist carbachol was shown to induce a short-term increase in firing rate, increase holding current and decrease input resistance of interpositus CN neurons. Carbachol also induced long-term depression of evoked inhibitory postsynaptic currents and a short-term depression of evoked excitatory postsynaptic currents. All effects were shown to be dependent upon muscarinic acetylcholine receptor activation. Overall, the present study has identified muscarinic receptor activation as a modulator of CN activity.

## Introduction

The cerebellum, a structure containing approximately 80% of neurons in the human brain [1], has well-established roles in motor functions including coordination, balance, postural control, the accurate execution of fine movements, and in association with such roles it is central to motor learning [2, 3]. The cerebellum has also been linked to a number of non-motor functions, including cognitive planning [4], spatial representation [5], fear responses [6], sensory processing [7], language [8] and attention [9]. This wide range of functions is likely due to connections—either direct or indirect—with a wealth of brain regions including, but not limited to, the motor cortex [10], basal ganglia [11], prefrontal cortex [12–14] and hippocampus [15, 16].

The cerebellar nuclei (CN) contain the final output neurons of the cerebellum. CN neurons receive excitatory collateral inputs from mossy fibres and climbing fibres, the two major synaptic inputs to the cerebellum, as well as inhibitory inputs from Purkinje cells, which form the sole output of the cerebellar cortex. In addition, there are several neuromodulatory inputs which project with varying patterns throughout the cerebellar cortex and CN. Despite early evidence for roles of the cholinergic system in modulating CN activity [e.g. 17] and extensive studies on this system in other brain regions, little is known about the roles of acetylcholine in the cerebellum.

Anatomical studies have demonstrated the presence of a set of varicose, beaded choline acetyltransferase (ChAT) positive fibres which form a diffuse network throughout the cerebellar cortex and CN [18]. Double labelling studies combining retrograde labelling and ChAT immunostaining have demonstrated these fibres arise from the pedunculopontine nucleus (PPN), laterodorsal tegmental nucleus and other brainstem nuclei including the lateral paragigantocellular nucleus, nucleus raphe obscurus, the vestibular nuclei and the pars interpolaris of the spinal trigeminal complex [18, 19]. The cholinergic influence arising from the PPN has recently been demonstrated physiologically in the CN, whereby electrical stimulation of the PPN produced a short latency (1.5–2 ms, implying a direct connection) orthodromic response in CN neurons which was blocked by acetylcholine receptor antagonists [20]. Whilst little is known about the functional role(s) of this projection it is suggested to be involved in sleep–wake cycle regulation as well as motor functions. Therefore, cholinergic inputs to the cerebellum may be involved in modulating intrinsic properties and synaptic inputs of CN neurons to mediate cerebellar activity in different behavioural states.

The cerebellum is a key player in motor learning with synaptic plasticity in cerebellar circuits, including the CN, thought to underlie the storage of information [21, 22]. As such, long-term synaptic plasticity has been demonstrated at both inhibitory inputs [23, 24] and excitatory inputs to CN neurons [25, 26]. The importance of acetylcholine receptors for learning and memory and its underlying synaptic plasticity mechanisms has been demonstrated in many brain regions both in vitro and in vivo [e.g. 27–29]. Similarly, in the cerebellum cholinergic receptor activation can modulate long-term potentiation at parallel fibre inputs to Purkinje cells in the cerebellar cortex [30], but nothing is known about how acetylcholine modulates plasticity within the CN. Acetylcholine may act to modulate neuronal activity and plasticity of synapses within CN, and through these mechanisms contribute to cerebellar function and learning. For example, in humans, systemic administration of the muscarinic acetylcholine receptor antagonist scopolamine impairs acquisition of cerebellar-dependent classical eyeblink conditioning [31, 32].

To study the effects of cholinergic receptor activation on synaptic and intrinsic properties of CN neurons, whole-cell patch clamp recordings were performed from interpositus nucleus neurons in rat cerebellar slices. The results provide evidence for a role of muscarinic acetylcholine receptors in mediating a number of effects in CN neurons. The modulatory influence on CN neurons, in turn, could alter their control of downstream brain regions and therefore impact cerebellar-mediated behaviours, including motor learning, through muscarinic receptor-dependent synaptic plasticity mechanisms.

## Methods

### Animals

All experiments were carried out in accordance with the UK Animals (Scientific Procedures) Act of 1986 and approved by the University of Bristol Animal Welfare and Ethical Review Body.

Male Wistar rats aged postnatal day 13–15 were obtained from breeding stocks at the University of Bristol. Animals were housed on a 12/12 h light/dark cycle (lights on at 8 a.m.) with the mother and littermates.

### Slice Preparation

Rats were deeply anaesthetised with isoflurane and decapitated. The cerebellum was rapidly removed and placed into ice cold (2–4 °C) sucrose cutting solution, containing (in mM): 189 sucrose, 10 d-glucose, 26 NaHCO_3_, 3 KCl, 5 MgSO_4_·7H_2_O, 0.1 CaCl_2_, 1.25 NaH_2_PO_4_, equilibrated with 95% O_2_/5% CO_2_. Coronal sections (400 µm thickness) containing the CN were prepared using ceramic blades on a vibrating microtome (7000smz, Campden Instruments, Loughborough). For the first 30 min following slicing, slices were kept in artificial cerebrospinal fluid (ACSF) containing (in mM): 124 NaCl, 26 NaHCO_3_, 3 KCl, 1.4 NaH_2_PO_4_, 1 MgSO_4_·7H_2_O, 10 d-glucose and 2 CaCl_2_ (equilibrated with 95% O_2_/5% CO_2_) at approximately 35 °C, and then at room temperature for at least another 30 min before recording. Following incubation slices were bisected along the midline.

### Whole Cell Recordings

Slices were placed in a submerged chamber and continuously perfused with ACSF at a rate of approximately 2 ml/min. Bath temperature was maintained at 32 ± 1 °C. Whole cell patch clamp recordings were made from neurons of the interpositus nucleus identified on an upright microscope (Eclipse E600 FN, Nikon Instruments) using a 40× water immersion objective and infrared differential interference contrast optics. The CN were identified based on their shape and location within the white matter, and large cells, corresponding to large glutamatergic projection neurons (soma > 20 µm), were targeted for recording [33].

Recordings were made using pipettes (2–5 MΩ) pulled from borosilicate glass capillaries on a horizontal puller (P-97, Sutter Instrument). Pipettes were filled with caesium-based internal solution for voltage clamp recordings (containing (in mM): 130 CsMeSO_4_, 10 HEPES, 0.5 EGTA, 4 MgATP, 0.3 NaGTP, 5 QX-314-Cl^−^, and 8 NaCl, titrated to pH 7.25 using CsOH, osmolarity 280–290 mOsM) and potassium-based solution for current clamp recordings (containing (in mM): 145 K-gluconate, 5 NaCl, 10 HEPES, 0.2 EGTA, 0.3 NaGTP and 4 MgATP, titrated to pH 7.25 using KOH, osmolarity 280–290 mOsM). Recording electrodes were placed in contact with the soma of a target neuron and after forming a tight seal (> 1 GΩ) negative pressure was applied using suction to achieve the whole cell configuration.

Spontaneous action potentials were recorded in current clamp configuration at resting potential (no current injection). In current clamp experiments, recordings commenced after obtaining whole cell configuration and compounds were bath applied in ACSF at times specified in the “Results” section.

Postsynaptic responses were evoked using a concentric bipolar stimulating electrode (FHC) placed in the white matter adjacent to the interpositus nucleus. Constant current pulses of 0.1 ms were delivered using a constant current isolated stimulator (DS3, Digitimer). Inhibitory GABAergic responses were examined during pharmacological blockade of excitatory transmission, by adding the AMPA receptor antagonist NBQX (5 µM) and the NMDA receptor antagonist D-AP5 (50 µM) to the ACSF. Excitatory glutamatergic responses were examined during pharmacological blockade of GABAergic transmission, by adding the GABA_A_ receptor antagonist picrotoxin (50 µM) to the ACSF. After obtaining stable response amplitude for at least 10 min, compounds were bath applied in the ACSF as specified in the “Results” section.

In voltage clamp recordings cells were held at − 50 mV for both inhibitory postsynaptic current (IPSC) and excitatory postsynaptic current (EPSC) recordings. Series resistance and input resistance were monitored at 30 s intervals throughout voltage clamp experiments by use of a 5 mV hyperpolarising step; data were discarded if series resistance deviated more than 20% from baseline. Data were acquired using an Axopatch 200B amplifier (Molecular Devices) and WinLTP software, filtered at 5 kHz and digitised at a sampling rate of 20 kHz for voltage clamp recordings and 40 kHz for current clamp recordings (Digidata 1322A, Molecular Devices). No correction was applied to compensate for liquid junction potential.

### Data Analysis

WinLTP [v1.10, 34] was used for online assessment of evoked response amplitude and monitoring series and input resistance of cells, and WinLTP reanalysis software was used for post-hoc experimental measurements. Spontaneous firing rate was analysed post-hoc using Spike2 (v7.12, CED). For spontaneous firing experiments data are plotted as z-score firing rate over time (firing rates for individual cells z-scored and then averaged, mean ± SEM). The difference between firing rate and membrane potential at 4–5 min following the start of drug application and baseline (the minute before drug application, to allow stabilisation of firing rate and membrane potential with dialysis of the internal solution) was calculated. Therefore, in these experiments a difference of 0 corresponds to no change in firing rate. Voltage clamp data are expressed as the mean ± SEM and are normalised to baseline values, with a baseline average of 1. To account for any antagonist effects baseline was the 10-min period preceding carbachol application. Graphs were produced using SigmaPlot (Windows Version 13.0).

### Statistics

All statistical analyses were performed using SPSS statistics software, as stated in the “Results” section. Spontaneous firing frequencies were analysed using the Wilcoxon singed ranks test (as data were not normally distributed) using raw firing rates before and during drug application. In voltage clamp experiments, measurements of holding current, input resistance and postsynaptic current amplitude during baseline were compared with the same measures at a set time point following drug application, as stated in the appropriate “Results” section, and tested for statistical significance using a two-tailed paired t-test of average values (before normalisation). Between group differences were assessed using a one-way ANOVA performed on normalised values.

## Results

### Cholinergic Receptor Modulation of Firing Rate

**Fig. 1:**
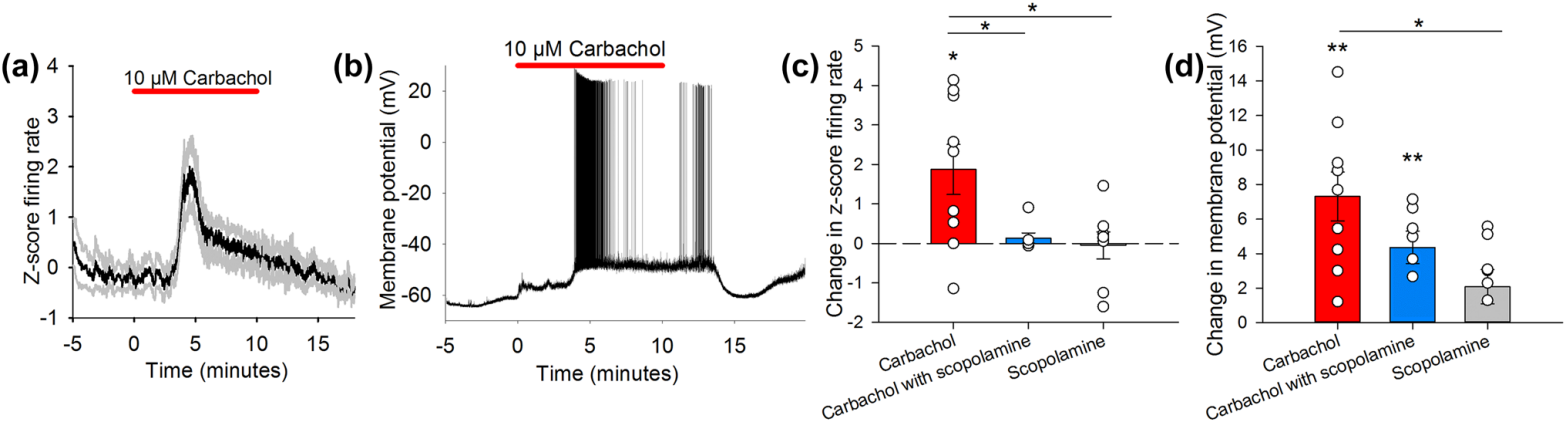
Effects of cholinergic receptor activation on the spontaneous firing rates and membrane potential of interpositus neurons in slices. **a** Bath application of carbachol increased the firing rate of interpositus neurons during whole cell recordings (n = 9). **b** Example experiment showing membrane depolarisation and increased firing rate in response to carbachol application. **c** Summary of difference in firing rate 4–5 min after the start of carbachol application, relative to pre-drug firing levels (compared using Wilcoxon signed rank test, *p < 0.05). The carbachol-induced increase in firing was blocked by scopolamine (n = 7) and scopolamine alone had no effect on firing rate (n = 8). **d** Change in membrane potential relative to pre-drug for the three conditions. Carbachol alone and carbachol with scopolamine significantly increased the membrane potential, but scopolamine alone had no significant effect (paired t-test **p < 0.01). Groups in **c** and **d** compared with one-way ANOVA, *p < 0.05. Data plotted as mean ± SEM

We first investigated the effect of a cholinergic agonist on CN neuron firing rates. Bath application of the cholinomimetic compound carbachol (10 µM) increased the firing rate of CN neurons from 4.13 ± 1.07 Hz to 8.54 ± 1.84 Hz (1.87 ± 0.63 difference in z-score firing rate at 4–5 min into carbachol application compared to the minute before carbachol; Fig. 1a, n = 9, Wilcoxon signed ranks test: z = − 2.100, p = 0.036). The coefficient of variance of the inter-spike time interval (CV) was 0.17 ± 0.05 during baseline and 0.62 ± 0.41 during carbachol application (Wilcoxon signed ranks test: Z = − 0.315, p = 0.752), and the CV2 was 0.09 ± 0.02 during baseline and 0.15 ± 0.05 during carbachol application (Wilcoxon signed ranks test: Z = − 0.943, p = 0.345).

To investigate the receptor subtypes involved in the carbachol-induced increase in firing rate, carbachol was co-applied with the broad-spectrum muscarinic receptor antagonist scopolamine (10 µM). The carbachol-induced increase in firing rate was blocked in the presence of scopolamine (0.13 ± 0.13 difference in z-score firing rate; Fig. 1c, n = 7, Wilcoxon signed ranks test: Z = − 1.069, p = 0.285). To test if there was any tonic activation of muscarinic receptors in the slice preparation, scopolamine was applied in the absence of carbachol. Scopolamine alone had no effect on firing rate (− 0.05 ± 0.34 difference in z-score firing rate, Fig. 1c, n = 8, Wilcoxon signed ranks test: Z = − 0.420, p = 0.674). The change in firing rate following carbachol was significantly different to both co-application with scopolamine and scopolamine alone (Fig. 1c, one-way ANOVA on difference in z-score with Tukey’s HSD post-hoc: F(2,21) = 5.557, p = 0.012; carbachol versus carbachol with scopolamine p = 0.040, carbachol versus scopolamine alone p = 0.017).

The membrane potential was depolarised in response to carbachol by an average of 7.31 ± 1.42 mV when comparing the minute before application to 4–5 min following the start of application (Fig. 1d, from − 51.19 ± 1.89 mV to − 43.88 ± 1.88 mV, n = 9, paired t-test: t(8) = − 5.153, p = 0.001). Co-application of scopolamine with carbachol reduced the difference in membrane potential following carbachol application to 4.36 ± 0.95, although a statistically significant increase still occurred (Fig. 1d, n = 7, paired t-test t(6) = − 4.608, p = 0.004). Scopolamine alone had no significant effect on membrane potential of CN neurons (Fig. 1d, 2.08 ± 0.99 mV, n = 8, paired t-test t(7) = − 2.096, p = 0.074).

Previous experiments have shown that activation of cholinergic receptors can increase CN firing rates in vivo [e.g. 17]. Our results show that acetylcholine receptors control firing rates in vitro in the absence of ongoing sensory inputs that would be present in vivo. Importantly, we show that the carbachol-induced increase in firing rate depends upon the activation of muscarinic acetylcholine receptors (summarised in Fig. 1c).

### Cholinergic Receptor Modulation of Holding Current and Membrane Resistance in Voltage Clamp

**Fig. 2:**
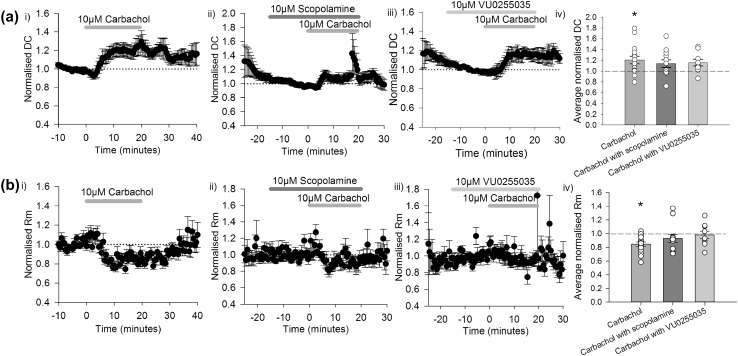
Acetylcholine receptor activation depolarised interpositus neurons and caused a decrease in input resistance. **a** (i) Carbachol application produced an increase in holding current required to voltage clamp interpositus neurons (n = 15) that was not significant in the presence of (ii) scopolamine (n = 12) or (iii) VU0255035 (n = 9). (iv) The average normalised current required to maintain membrane potential at − 50 mV in the last 10 min of carbachol application compared to the 10 min prior to carbachol application. **b** (i) Input resistance (Rm) of interpositus neurons decreased in response to carbachol application (n = 15). This effect was reduced by (ii) scopolamine (n = 12) or (iii) VU0255035 (n = 9). (iv) The average normalised input resistance in the last 10 min of carbachol application compared to the 10 min prior to carbachol application. *p < 0.01, paired t-test comparing baseline and last 10 min of carbachol. Data plotted as mean ± SEM

Carbachol (10 µM) application produced an increase in the holding current required to voltage clamp neurons at − 50 mV (1.21 ± 0.07 during the last 10 min of carbachol application relative to baseline, Fig. 2ai; n = 15; paired t-test: t(14) = 3.153, p = 0.007). The holding current was not significantly different when carbachol was co-applied with scopolamine (10 µM, 1.14 ± 0.07 relative to 10 min period prior to carbachol application; Fig. 2aii, n = 12, paired t-test: t(11) = 0.988, p = 0.344). The carbachol-induced changes in holding current were reduced when the M_1_ selective antagonist VU0255035 was co-applied with carbachol, and the increase was no longer statistically significant (1.16 ± 0.06, Fig. 2aiii, n = 9, paired t-test: t(8) = 1.726, p = 0.123). A summary of the effects on holding current during the last 10 min of carbachol application relative to baseline is shown in Fig. 2aiv.

A decrease in input resistance occurred with carbachol application (0.85 ± 0.03 relative to baseline; Fig. 2bi; n = 15, paired t-test: t(14) = 5.298, p = < 0.001) but this was inhibited when carbachol was co-applied with scopolamine (0.93 ± 0.06; Fig. 2bii, n = 12, paired t-test: t(11) = 1.644, p = 0.129). There was also no significant change in input resistance when VU0255035 was co-applied with carbachol (0.98 ± 0.05, Fig. 2biii, n = 9, paired t-test: t(8) = 0.304, p = 0.769). The results summarised in Fig. 2aiv Fig. 2biv show that muscarinic receptors, including M_1_ receptors, contribute to carbachol-induced changes in membrane potential and input resistance.

### Cholinergic Receptor Modulation of Postsynaptic Currents

**Fig. 3:**
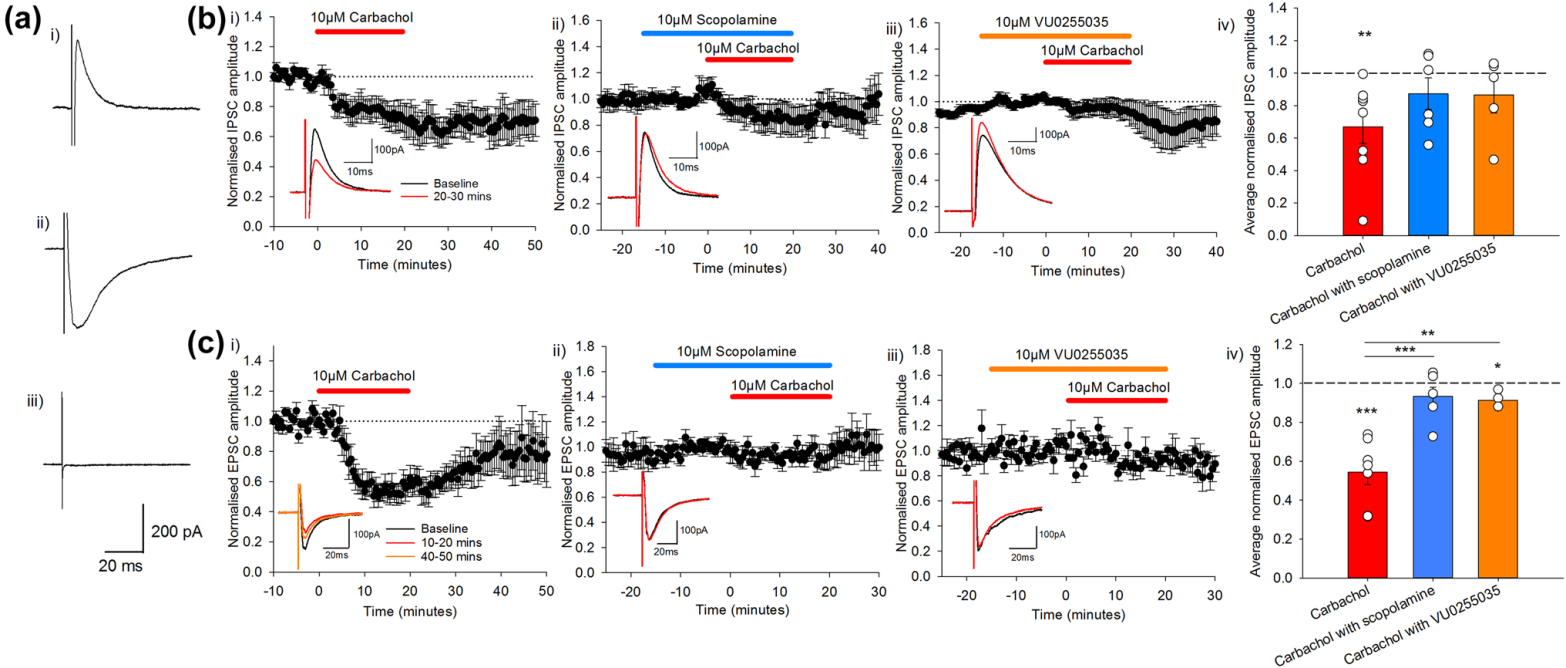
Effects of cholinergic receptor activation on synaptic responses within the interpositus nucleus. **a** Synaptic responses recorded from interpositus neurons following electrical stimulation of the adjacent white matter: (i) Example IPSC recorded in the presence of NBQX (5 µM) and D-AP5 (50 µM); (ii) Example EPSC recorded in the presence of picrotoxin (50 µM); (iii) all components of the synaptic response were blocked by the co-application of picrotoxin, NBQX and D-AP5. All traces shown are an average of ten consecutive responses; holding potential − 50 mV. **b** Effects of the following cholinergic compounds on inhibitory synaptic responses within interpositus neurons were investigated: (i) Carbachol (10 µM, n = 8); (ii) the muscarinic antagonist scopolamine co-applied with carbachol (10 µM, n = 6); and (iii) the M_1_ antagonist VU0255035 co-applied with carbachol (10 µM, n = 5); (iv) Average IPSC amplitude in the 10 min following the end of drug application relative to baseline. **c** Effects of the following compounds on excitatory synaptic responses within interpositus neurons were investigated: (i) Carbachol (10 µM, n = 7); (ii) scopolamine co-applied with carbachol (10 µM, n = 6); and (iii) VU0255025 co-applied with carbachol (10 µM, n = 4); (iv) Average EPSC amplitude during the last 10 min of drug application relative to baseline. Paired t-test for baseline vs carbachol comparison; one-way ANOVA for between groups comparison, *p < 0.05, **p < 0.01, ***p < 0.001. Average values normalised to the baseline of each experiment are plotted ± SEM. Insets show example traces (average of 20 consecutive responses from a 10-min period) displayed from the baseline (black) and from selected time-points following drug application as indicated in **b** and **c**

The white matter surrounding the CN comprises a combination of inhibitory GABAergic Purkinje cell axons and excitatory glutamatergic mossy fibres and climbing fibres. Under control conditions electrical stimulation of the white matter resulted in both inhibitory and excitatory postsynaptic responses of a similar latency in interpositus neurons recorded in voltage clamp. In the following experiments inhibitory and excitatory components were studied individually following their pharmacological isolation (Fig. 3a). Inhibitory responses were isolated through bath application of the AMPA receptor antagonist NBQX (5 µM) and the NMDA receptor antagonist D-AP5 (50 µM, Fig. 3ai). Excitatory responses were isolated through bath application of the GABA_A_ receptor antagonist picrotoxin (50 µM, Fig. 3aii). Combined application of NBQX, D-AP5 and picrotoxin blocked all postsynaptic currents (Fig. 3aiii).

To investigate effects of cholinergic receptor activation on inhibitory synaptic inputs to the interpositus nucleus, carbachol was applied to the slice during IPSC recordings. Bath application of carbachol (10 µM) for 20 min decreased the average amplitude of IPSCs to 0.67 ± 0.10 relative to the baseline in the 10 min following the end of drug application (Fig. 3bi, n = 8, paired t-test: t(7) = 3.619, p = 0.009). This depression did not recover during the remainder of the recording period (repeated measures ANOVA comparing baseline to 10 min following end of carbachol application to 40–50 min time point: F(2,12) = 6.690, p = 0.011; baseline vs 40–50 min p = 0.046).

Scopolamine (10 µM) was bath applied before and during carbachol application (Fig. 3bii). In the 10 min following the end of drug application scopolamine reduced the effect of carbachol (average response 0.87 ± 0.10 compared to 10 min period prior to carbachol application, n = 6, paired t-test: t(5) = 1.171, p = 0.294). These data show that the carbachol-mediated depression involved activation of muscarinic acetylcholine receptors. To further investigate the muscarinic receptors involved, the M_1_ receptor specific antagonist VU0255035 (10 µM) was used. VU0255035 also reduced the effects of carbachol (0.87 ± 0.11 in the 10 min following the end of carbachol application; Fig. 3biii, n = 5, paired t-test: t(4) = 1.148, p = 0.315). The results, summarised in Fig. 3biv, show that although some changes in IPSC amplitude occurred in the presence of these antagonists, the effects of carbachol were reduced and therefore the depression observed in response to carbachol application is at least partly dependent upon M_1_ muscarinic receptor activation.

Carbachol was also applied to slices during EPSC recordings to investigate the effects of cholinergic receptor activation on excitatory synaptic inputs to the interpositus nucleus. Bath application of carbachol (10 µM) for 20 min decreased the amplitude of EPSCs, with peak depression occurring during the last 10 min of drug application (Fig. 3ci, n = 7). The average normalised response during this period was 0.54 ± 0.06 relative to baseline (paired t-test: t(6) = − 7.365, p < 0.001). A one-way repeated measures ANOVA comparing response amplitude during baseline, the last 10 min of drug application (peak depression), and post-drug application (40–50 min, to test long-term effects) showed a significant difference between baseline and the last 10 min of drug application, but not between baseline and the post-drug time point, showing that the effect was not long-lasting (test of within subject effects: F(2,12) = 5.129, p = 0.025; baseline vs. last 10 min of drug application mean difference = − 136.82, p = 0.001; other pairwise comparisons not significant; Bonferroni correction applied).

Scopolamine was bath applied prior to co-application of carbachol and inhibited the carbachol induced depression of EPSC amplitude (Fig. 3cii); in the last 10 min of carbachol application the average response was 0.93 ± 0.05 (n = 6, paired t-test: t(5) = − 1.342, p = 0.237). Similarly, co-application of VU0255035 reduced the effects of carbachol (0.91 ± 0.02) although a statistically significant depression compared to baseline was still observed (Fig. 3ciii, n = 4, paired t-test: t(3) = − 3.913, p = 0.030). The EPSC amplitude during the last 10 min of carbachol application was significantly different to that during either scopolamine or VU0255035 co-application (Fig. 3civ, one-way ANOVA with Tukey’s HSD post-hoc: F(2,14) = 16.545, p < 0.001; carbachol vs. carbachol with scopolamine p < 0.001; carbachol vs. carbachol with VU0255035 p = 0.002). This suggests that M_1_ muscarinic receptors are involved in carbachol-induced depression at excitatory inputs to CN neurons.

## Discussion

Our results demonstrate that muscarinic receptor activation in cerebellar slices contributes to several effects in CN neurons, including: (i) a brief increase in firing rate; (ii) a decrease in input resistance; (iii) long-term depression of inhibitory synaptic responses; and (iv) a shorter-term depression of excitatory synaptic responses. Most of these effects were reduced, although not always completely blocked, by either the broad-spectrum muscarinic antagonist scopolamine or the specific M_1_ antagonist VU0255035 (Fig. 2). This suggests that other receptors, such as nicotinic acetylcholine receptors, may be involved in mediating the full extent of carbachol-induced changes in CN neurons.

Following carbachol application the enhanced firing rate returned over time to baseline levels (Fig. 1). In the example neuron shown in Fig. 1b, carbachol induced a depolarisation of the neuron accompanied by action potential firing; as carbachol was washed from the bath the membrane returned to a more hyperpolarised potential and firing ceased. The pooled data show that on average the firing rate peaked and then returned to baseline levels during carbachol application (Fig. 1a). It may be that acetylcholine receptors had become desensitised to prolonged carbachol application. Scopolamine alone did not completely block the carbachol-induced increase in membrane potential (Fig. 1d), suggesting an involvement of nicotinic receptors which are prone to desensitisation following prolonged agonist application [35].

Depolarisation of CN neurons accompanied the observed increase in firing rate; carbachol induced a significant change in membrane potential of CN neurons in current clamp recordings (Fig. 1d). A sustained increase in the amount of current required to voltage clamp neurons at − 50 mV was also observed (Fig. 2ai), reinforcing the depolarising effects of carbachol. The magnitude of this depolarisation was reduced in the presence of scopolamine (Fig. 1d). Although scopolamine did not block the effects of carbachol on membrane potential—there was still a significant difference compared to baseline—the reduction appeared sufficient to prevent significant changes in firing, as no change in firing rate occurred during co-application of scopolamine with carbachol (Fig. 1c). This suggests that additional receptors unaffected by scopolamine (i.e. nicotinic receptors) contribute to the change in membrane potential, but that inhibiting muscarinic receptors is enough to prevent changes in firing rate in response to carbachol.

Synaptic inputs were not blocked during current clamp experiments, and so presynaptic effects of carbachol on the effects observed cannot be ruled out based on the current data. However, related changes in intrinsic properties were seen in voltage clamp experiments (increased holding current and decreased membrane resistance, Fig. 2) when synaptic blockers were present, suggesting that at least some of the effects of carbachol are mediated postsynaptically. Further experiments are needed to determine whether the effects of carbachol on synaptic currents (Fig. 3) are mediated through pre- or postsynaptic mechanisms.

The changes to membrane potential and input resistance following carbachol application (Fig. 2) are likely due to the modulation of several ionic conductances following muscarinic receptor activation. Muscarinic depolarisation typically shows an initial large inward current resulting from a decrease in inward potassium current and increase in hyperpolarisation-activated cation current Ih (M_3_ receptor mediated), and a later M_1_ receptor mediated decrease in inward potassium current [36]. This is usually associated with an increased input resistance; however, the experiments here displayed a decreased input resistance (Fig. 2bi). It has previously been observed in neurons of the basolateral complex of the amygdala that carbachol induced a depolarisation accompanied by decreased or unchanged input resistance, and this effect occurred during blockade of the M-current [37]. An alternative conductance pathway may therefore be related to these effects of carbachol, for example a calcium-dependent potassium or non-specific cation conductance, which may be similar to the present experiments [37, 38].

We observed different temporal profiles of depression of inhibitory and excitatory synaptic transmission within CN (Fig. 3bi, ci). The inhibitory responses recorded in CN neurons (e.g. Fig. 3ai) likely correspond to inputs from Purkinje cells. Purkinje cell projections are estimated to account for 70% of the innervation of the CN [39], and electrical stimulation of the white matter in the presence of glutamate receptor antagonists has been shown to generate a comparable response to that evoked from selective optogenetic activation of Purkinje cell axons [40], suggesting minimal contribution from other sources of inhibitory activity. The excitatory responses (e.g. Fig. 3aii) are likely to correspond to mossy fibre inputs; a study assessing mossy fibre and climbing fibre inputs to CN in young (postnatal day 12–23) mice selectively using optogenetic methods concluded that electrical stimulation in slices primarily recruits mossy fibre responses, as short term depression of EPSCs following repeated electrical stimulation is similar to that following selective mossy fibre activation, yet less than following selective climbing fibre activation [41, 42].

The relative strength and timing of excitation and inhibition of CN neurons may be key to information coding and storage in the cerebellum; the direction of long-term plasticity at mossy fibre-CN synapses depends on the state of the postsynaptic CN neuron [25, 26, 43]. Inhibition driven by Purkinje cells guides the strengthening of excitatory synapses to CN neurons by driving their hyperpolarisation, and instead causes depression if there is a reduced level of inhibition. The net effect acetylcholine on CN may be to produce a short-term increase in CN output that results from a long-term depression of inhibition (Fig. 3bi) but only short-term depression of excitatory inputs (Fig. 3ci). The changes in the strength and balance of excitatory and inhibitory inputs by acetylcholine receptor activation may therefore influence other plasticity mechanisms within the CN, and the resulting changes in cerebellar output signals via CN may affect downstream brain regions and thus cerebellar-mediated behaviours including motor learning.
